# Doping Profiles in Ultrathin Vertical VLS-Grown InAs Nanowire MOSFETs with High Performance

**DOI:** 10.1021/acsaelm.1c00729

**Published:** 2021-11-19

**Authors:** Adam Jönsson, Johannes Svensson, Elisabetta Maria Fiordaliso, Erik Lind, Markus Hellenbrand, Lars-Erik Wernersson

**Affiliations:** † Department of Electrical and Information Technology, Lund University, Box 118, 221 00 Lund, Sweden; ‡ National Centre for Nano Fabrication and Characterization, Technical University of Denmark, Fysikvej 307 & 126, 2800 Kongens Lyngby, Denmark; § Department of Material Science & Metallurgy, University of Cambridge, 27 Charles Babbage Road, CB3 0FS Cambridge, United Kingdom

**Keywords:** III−V, doping, electron holography, MOSFET, nanowire, InAs, VLS growth

## Abstract

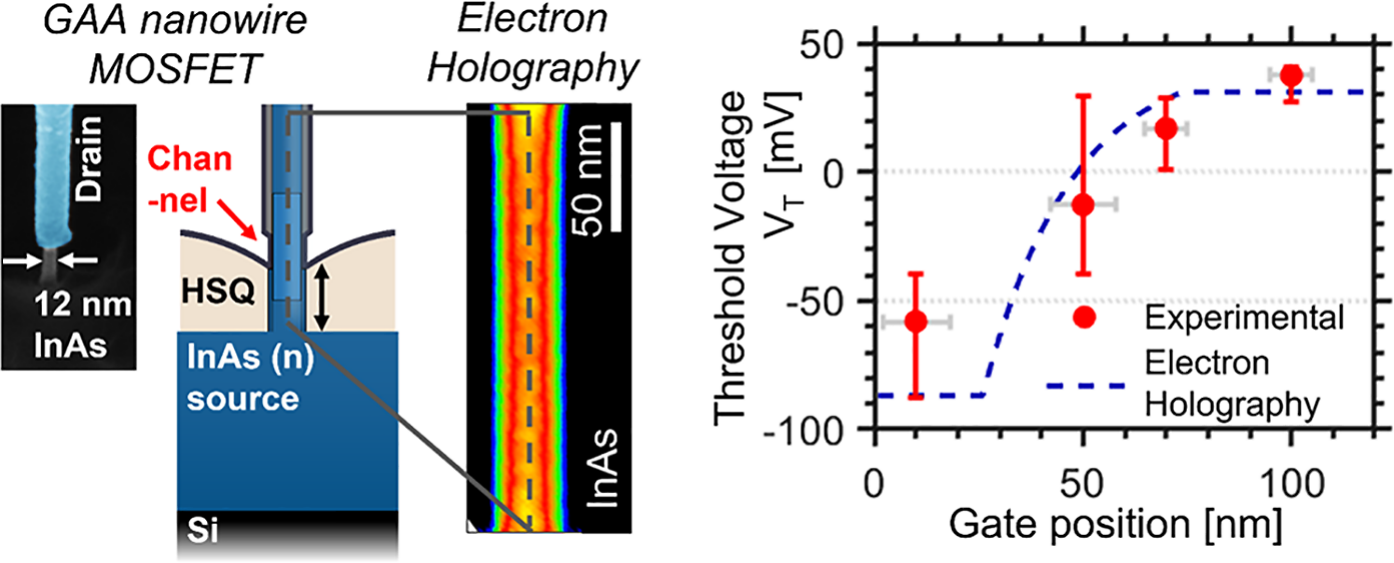

Thin vertical nanowires based on III–V compound semiconductors are viable candidates as channel material in metal oxide semiconductor field effect transistors (MOSFETs) due to attractive carrier transport properties. However, for improved performance in terms of current density as well as contact resistance, adequate characterization techniques for resolving doping distribution within thin vertical nanowires are required. We present a novel method of axially probing the doping profile by systematically changing the gate position, at a constant gate length *L*_g_ of 50 nm and a channel diameter of 12 nm, along a vertical nanowire MOSFET and utilizing the variations in threshold voltage *V*_T_ shift (∼100 mV). The method is further validated using the well-established technique of electron holography to verify the presence of the doping profile. Combined, device and material characterizations allow us to in-depth study the origin of the threshold voltage variability typically present for metal organic chemical vapor deposition (MOCVD)-grown III–V nanowire devices.

## Introduction

Vertical III–V nanowires (NWs) provide new capabilities in many semiconductor device technologies such as light-emitting diodes,^1^ solar cells,^2,3^ and improved complementary metal-oxide semiconductor (CMOS) transistor architectures attractive beyond the scaling limit for the conventional Si technology.^4−8^ Thin nanowires allow for greater flexibility in material integration due to their smaller footprint, which enables a larger lattice mismatch by radial strain relaxation. The possibility of accommodating larger lattice mismatch is a key benefit for bottom-up integration of nanowires on various substrates as well as for forming heterostructures within the nanowire to an extent not possible in planar technologies.^9,10^ However, adequate control of doping levels and gradients within the nanowire is essential for both low contact and access resistances and also for enabling good electrostatics at scaled dimensions. Therefore, new and device-specific characterization methods are critical to support further development of thin vertical III–V nanowire devices.

Vapor–liquid–solid (VLS) epitaxial growth from Au particles enables the formation of high-aspect-ratio nanowires suitable for device implementation. Nanowire doping is typically introduced in situ during nanowire growth, and incorporation can occur both axially through the catalyst particle and radially on the nanowire sidewalls.^11^ The amount of axial doping within the nanowires is highly affected by dopant species accumulating in the metal catalyst, typically creating a reservoir effect. Namely, dopants species remain within the gold particle independent of growth chamber conditions. The formation of an abrupt doping profile is therefore challenging, and the optimal conditions will depend on dopant solubility in the particle, particle size, and growth rate. However, using VLS enables relatively low temperature during epitaxial growth (<460° for InAs), which serves to suppress diffusion of dopant species already incorporated within the crystal.^12^ Particularly, Sn is often used to achieve high n-type carrier concentration in InGaAs, up to 5 × 10^19^ cm^–3^. However, Sn is an amphoteric dopant resulting in compensational doping at high concentrations.^13^ VLS growth of GeSn nanowires has demonstrated Sn incorporation well in excess of equilibrium solubility in bulk Ge. For InAs nanowires specifically, the Sn doping is typically below the detection limit of energy-dispersive X-ray (EDX) analysis-based methods, indicating incorporation under equilibrium solubility.^14^

Efficient characterization with spatial resolution of the doping within a nanowire remains challenging due to its small geometry. For Au-catalyzed VLS growth, the reservoir effect reportedly leads to a doping grading length on the order of the nanowire diameter.^15^ Traditional methods, used for planar films, such as Hall measurements have been applied to large nanowires (diameter >100 nm), but are not applicable to thin nanowires required for transistor applications.^16−18^ In addition, capacitance-based measurements have typically been applied to gated nanowires in large nanowire arrays for the evaluation of the doping level, although geometrical limitations such as large surface-to-volume ratio and parasitic capacitances, as well as dynamic carrier interaction at oxide traps present within the gate-stack restrict the measurement accuracy.^19^ Approaches that allow sufficient spatial resolution are limited to atom probe tomography and electron holography. Specifically, electron holography offers sensitivity to active doping via the built-in potential they generate, while also maintaining a spatial resolution in the nanoscale range.^20−22^ However, these methods require separately prepared samples for characterization of thin nanowires used within devices.

**Figure 1:**
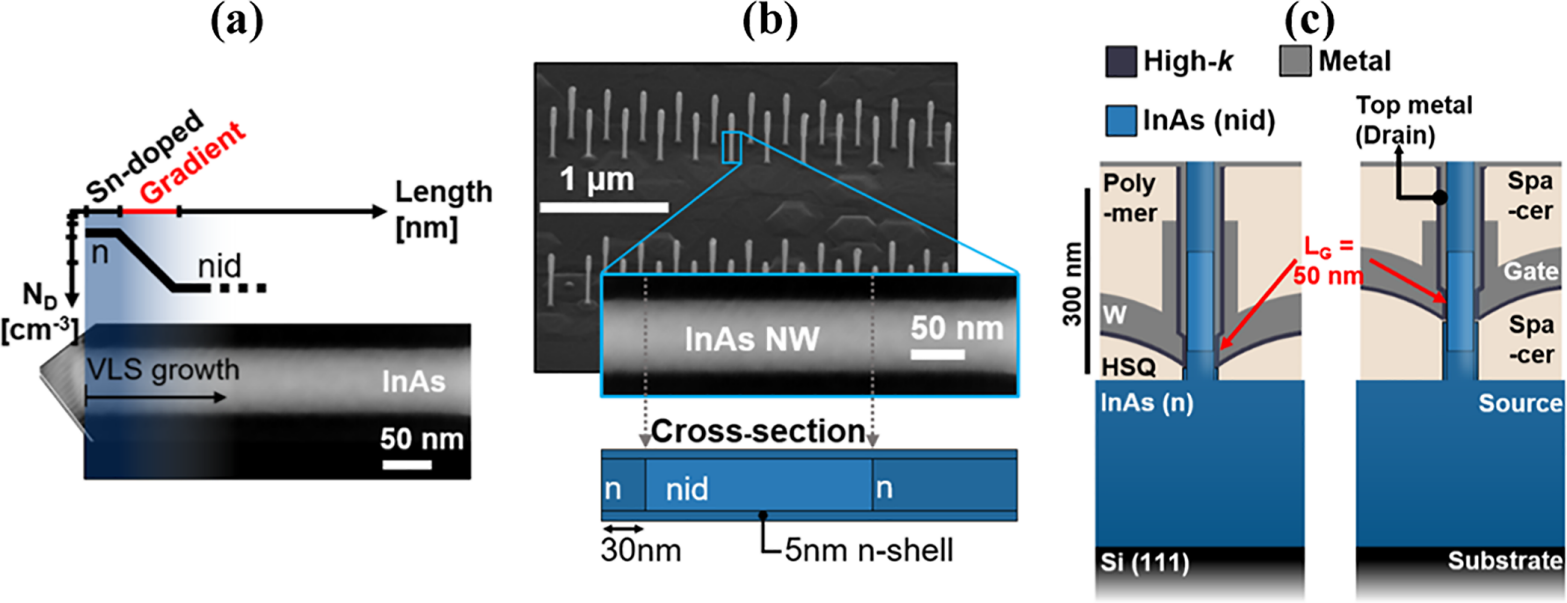
(a) TEM image of the implemented InAs nanowires, highlighting the expected doping gradient induced by in situ Sn doping during metal-seeded VLS growth. (b) Schematic representation of the doping profile as estimated from growth conditions and geometry (Nanowire Growth section) (c) Schematic representation of the finalized vertical nanowire MOSFET with varied gate position retaining a constant *L*_g_ of 50 nm.

In this paper, we present a novel characterization method used to probe the axial doping distribution in high-performance and scaled (gate length = 50 nm) metal−oxide−semiconductor field-effect transistors (MOSFETs) by varying the gate position along a vertical InAs nanowire and evaluating the resulting threshold voltage (*V*_T_) shift. We apply this method to vertical III–V nanowire-based MOSFETs with thin diameters (12 nm) and state-of-the art performance. Particularly, we systematically study the correlation between the threshold voltage and the varying doping distribution in the axial direction of the incorporated nanowires. The *V*_T_-based characterization method is verified by employing well-established electron holography measurements to independently characterize the axial doping gradient along the nanowire by electrostatic potential. Electron holography thus allows us to evaluate the extent of the reservoir effect prevalent for VLS growth by characterizing the doping distribution gradient; see Figure 1a. The growth results are further characterized by transmission electron microscopy (TEM) imaging, which derives the nanowire crystal structure. The fabricated n-type III–V (InAs channel) transistors exhibit very high transconductance (up to 2.6 mS/μm) and low access resistance (down to 300 Ω μm) attributed to the introduction of a doped segment in the bottom of the nanowire as well as implementing core–shell nanowires (radial shell growth). Furthermore, the doping profile is correlated with actual transistor performance metrics for method validation.^23^ Previous studies of *V*_T_ shifts in III–V MOSFETs have typically been attributed to quantum size effects for homogeneously doped FinFETs.^24−26^ Here, we characterize the axial doping gradient by*V*_T_ shift for ultrathin vertical gate-all-around (GAA) nanowire MOSFETs by a novel method enabled by advanced, high-precision, fabrication techniques. Furthermore, this study concludes that the doping gradient within the nanowire provides the main contribution to the *V*_T_ shift.

## Experimental Section

The implemented InAs nanowires are grown from Au dots (32 nm diameter), defined by electron beam lithography (EBL).^5,27^ The nanowire VLS growth is performed on a substrate consisting of a 260 nm epitaxially grown n-doped InAs layer on top of a p-type Si(111) substrate. The highly conductive InAs layer facilitates low access resistance and provides an easy path for device isolation by mesa etching of the layer, which are benefits compared to other growth approaches where the nanowires are directly integrated on the Si substrate.^28,29^ Sn is used for doping the top and bottom part of the InAs to reduce access resistance, while the intermediate section of the nanowire is not intentionally doped; see Figure 1b. The expected doping profile can be predicted based on geometry and growth conditions, where the gradient from the Sn-doped bottom segment is estimated to be in the order of the gold particle size, in our case about 30 nm (see the Methods section for detailed growth parameters).^15^ The nanowires are also radially overgrown with a 5 nm n-doped InAs shell (Figure 1b), which contributes to improved contact resistance for the final devices.^30^ Vertical GAA MOSFET devices are formed by following a self-aligned gate last process. This allows selective recess etching of the gate region, which enables nanowire diameters of only 12 nm and further minimizes the drain resistance using a wrap-around drain contact with a gate overlap (detailed description is found in the Device Fabrication section), as illustrated in Figure 1c. We observe that the recess etching leaves a highly doped InAs shell as well as a metal contact adjacent to the source and drain, respectively, which serves to mitigate access resistance further. The fabrication method allows for varied gate positions while retaining a constant gate length of 50 nm. A hexagonal double-row array structure consisting of 184 nanowires is used for each MOSFET, where an internanowire pitch of 300 nm is implemented to minimize proximity effects during VLS growth such as material diffusion.^31^ The double-row layout with multiple nanowires provides sufficient absolute current and transconductance for the fabricated devices, which enables high-frequency measurements. The use of multiple nanowires in each device also provides beneficial averaging with respect to process conditions within the array, which serves to suppress unwanted variations of electrical properties for similar devices.^32^

## Results

**Figure 2:**
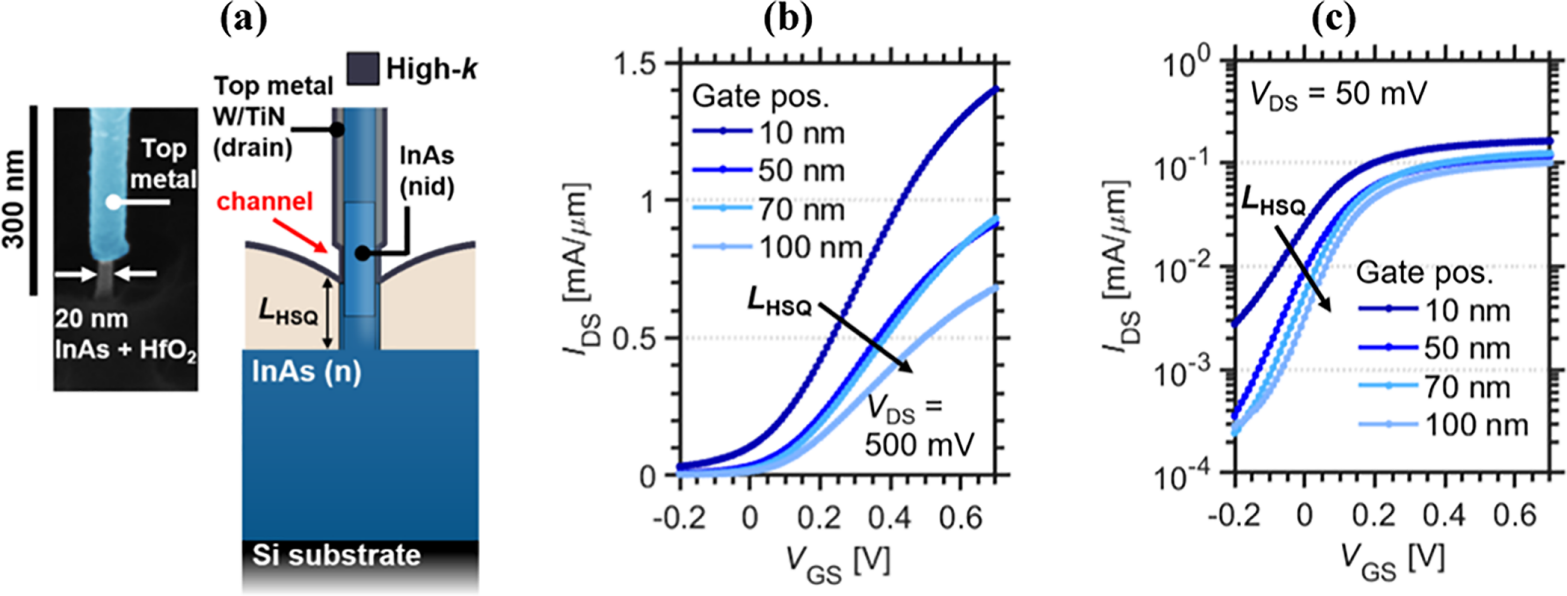
(a) Schematic representation of the nanowire prior to gate deposition, where the thickness of the HSQ spacer defines the position of the gate. (b) Transfer characteristics (linear scale) at *V*_DS_ = 500 mV representing the on-state for varied gate position *L*_HSQ_. (c) Transfer characteristics (log scale) at *V*_DS_ = 50 mV representing the off-state for varied gate position *L*_HSQ_.

Device schematics and representative transfer characteristics for MOSFETs with varying gate position along the vertical nanowire are presented in Figure 2 (see expanded dataset in the Supporting Information). The selective etching of the channel region enables the formation of a thin nanowire channel diameter of 12 nm, which is necessary to suppress short channel effects at short gate lengths (*L*_g_ = 50 nm); see Figure 2a.^25^ A key process step in our method is that we systematically vary the thickness of the bottom hydrogen silsesquioxane (HSQ) spacer (*L*_HSQ_) between the MOSFETs, which translates to a shifted gate position along the nanowires. Therefore, based on the HSQ thickness *L*_HSQ_, different parts of the nanowires are covered by the gate and, in effect, this realizes MOSFETs with different doping profiles. Thus, when moving the gate position upwards along the nanowires, the doping within the channel will gradually shift from high doping level (n^+^) close to the bottom segment toward nonintentional doping (nid), a change that profoundly influences the transistor metrics. This behavior is evident from the transfer characteristics at *V*_DS_ = 500 mV (Figure 2b), which exhibit improved drive current when the gate is placed within the highly doped region, thus leaving no ungated resistive regions at the source side.^33^ In addition, the transfer characteristics at the lower drain bias, at *V*_DS_ = 50 mV (Figure 2c), demonstrate a systematic shift from depletion (*V*_T_ < 0 V) toward enhancement mode (*V*_T_ > 0 V) operation between the devices, as well as improved modulation for elevated gate position. Both effects can be attributed to the variation in the channel carrier concentration. Notably, these devices are processed in parallel on the same sample, removing potential variation due to, for instance, processing and deposition conditions.

**Figure 3:**
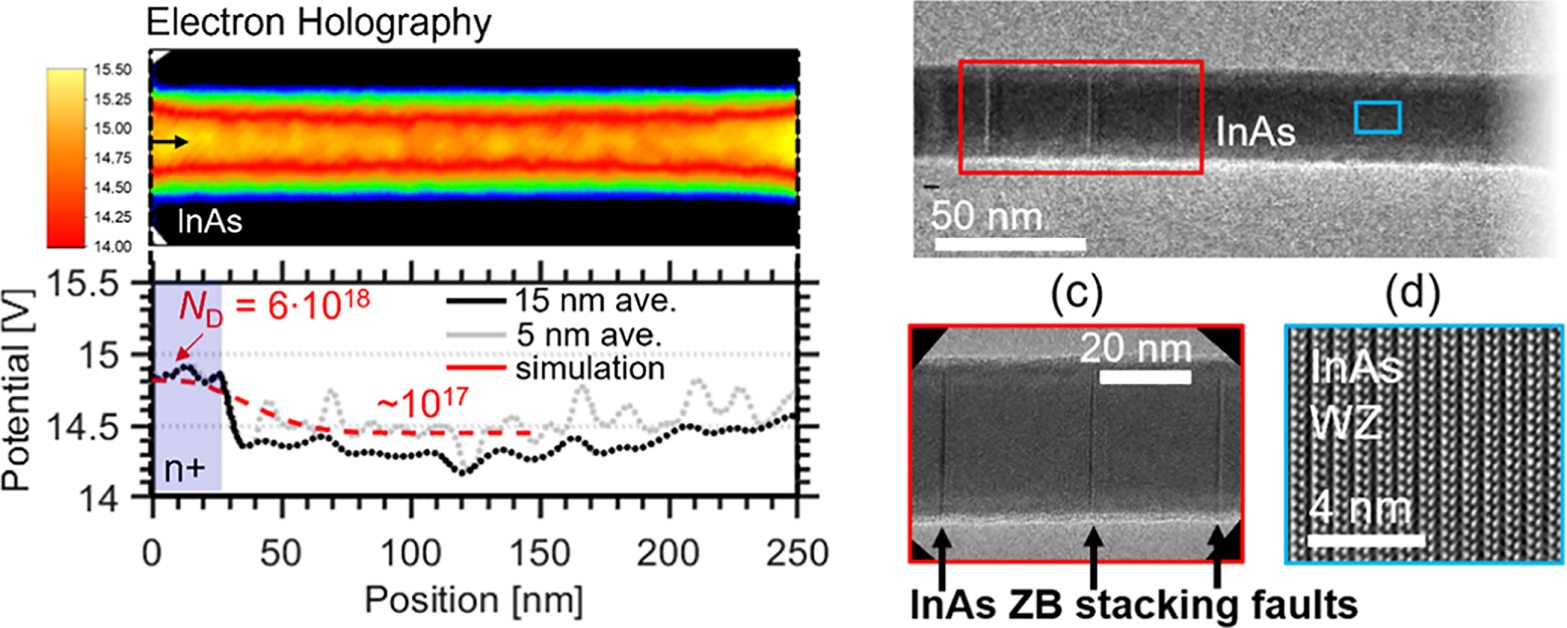
(a) Phase map and axial potential calculated from electron holography data for the InAs nanowire showing the expected initial n^+^-region (0 to ∼25 nm). Axial potential extracted from the center core (mean value of 15 nm, and 5 nm wide volume) of the nanowire phase map, where oscillations are caused by diameter variation. The simulated potential is calculated using a 1D zero-current model to determine the self-consistent electrostatic potential. The observed potential variation is significantly larger than the measurement error of ±0.092 V. (b) High-resolution TEM image of the heterostructure nanowire showing (c) stacking faults at regions with higher Sn doping incorporation and (d) WZ crystal structure of the InAs segment.

By applying electron holography and TEM imaging to a nanowire with the same growth conditions (albeit 44 nm diameter Au dot) the axial doping distribution and crystal structure of the InAs nanowires can be evaluated; see Figure 3. Here, separate samples are prepared where the InAs radial shell is etched after growth to analyze the properties of the core InAs segment (Figure 1b); see the TEM Analysis section. Using electron holography, a phase map is constructed and translated to electrostatic potential (details in the TEM Analysis section) (Figure 3a).^34^ The technique is here used to assess the built-in potentials (*V*_BI_) and the active doping in the radial and axial directions of the nanowires. The axial potential is calculated as the mean value of 5 nm wide and 15 nm wide volumes, respectively, along the center of the nanowire phase map, to further validate the elevated potential (0 to ∼25 nm) at the beginning of the InAs nanowire section. Electron holography techniques are also highly diameter-dependent, which contribute to the observed oscillations within the data, where many of the oscillations can be attributed to zincblende stacking faults along the nanowire. Fitting a modeled electrostatic potential, based on a 1D zero-current model (1D Poisson-solver) to the measured potential demonstrates the highest doping concentration *N*_D_ from 6 × 10^18^ cm^–3 ^^35^ at the bottom of the nanowire down to about 6 × 10^17^ cm^–3^ in the intrinsic segment. Here, an exponentially decaying gradient corresponding to the nanowire Au dot diameter is assumed, which corresponds to a decay of the doping concentration of 44 nm/decade.^15^ High-resolution TEM imaging is also performed on the same type of nanowire, visualizing the wurtzite crystal structure with zincblende stacking faults. As expected, the zincblende stacking faults are prevalent for the InAs segment with a higher level of Sn doping incorporation.^9^

**Figure 4:**
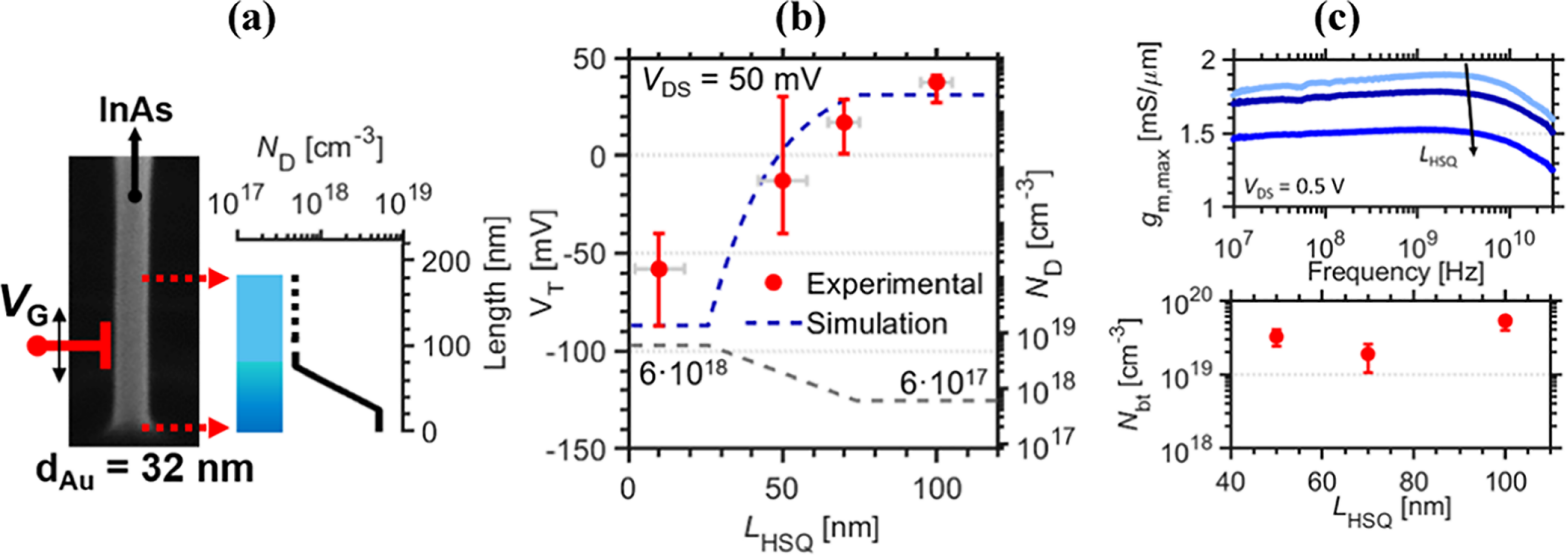
(a) Doping gradient achieved by electron holography varying from 6 × 10^18^ down to 6 × 10^17^ cm^–3^ with an exponential decay of 44 nm/decade. (b) Measured threshold voltage *V*_T_ dependence of gate position *L*_HSQ_ and a theoretical model considering the calculated doping gradient from electron holography. *V*_T_ is extracted at the linear mode of operation (*V*_DS_ = 50 mV) to suppress short channel effects. Five devices are measured for each gate position at *L*_HSQ_ < 90 nm and two devices for *L*_HSQ_ = 100 nm. The theoretical simulation considers fully doped cylindrical junctionless transistors.^36^ (c) Frequency behavior of maximum transconductance *g*_m,max_ and oxide trap density derived from *g*_m,max_ vs frequency dispersion for varying gate position. *N*_bt_ is estimated by considering boarder traps responding within a region of 10^8^–10^9^ Hz corresponding to an oxide depth of about 0.1–0.3 nm when assuming elastic tunneling.^38^

Threshold voltage *V*_T_ was calculated from the measured transfer characteristics, and frequency-dependent measurements were carried out to verify the negligible influence of gate oxide defects *N*_bt_ for varying gate position along the vertical nanowire MOSFET. The results are presented in Figure 4, where a schematic image is also provided that represents the axial doping distribution of the nanowire determined via electron holography (Figure 4a). Figure 4b presents *V*_T_ versus *L*_HSQ_, where *V*_T_ is calculated by extrapolating from maximum transconductance at *V*_DS_ = 50 mV. Here, five devices are measured for each gate position at *L*_HSQ_ < 90 nm as well as two devices at*L*_HSQ_ = 100 nm. The *V*_T_ shifts by a total of about 100 mV as the gate position is systematically moved from the bottom to the upper part of the nanowire. The threshold voltage can be described (dashed line) by an analytical model for doped junctionless GAA nanowires MOSFETs as *V*_T_ = *V*_fb_ – *qN*_D_*K*, where *V*_fb_ and *N*_D_ represent the flatband voltage and channel doping concentration, respectively. Furthermore, *K* = *r*^2^(4ε_r_)^−1^ ln(1 + *t*_ox_/*r*)*r*^2^(64ε_r_)^−1^ summarizes different scaling parameters, where *t*_ox_ is the thickness of the gate oxide, *r* is the nanowire radius, and ε_s_ and ε_r_ represent the relative permittivities of the semiconductor and gate oxide, respectively.^36^ This theoretical model is applied by considering the electron holography results, including a difference of an order of magnitude (6 × 10^18^ to 6 × 10^17^ cm^–3^) and a 44 nm/decade decay in doping concentration along the axial direction (Figure 3a). The model describes well the transition in *V*_T_ both in magnitude and position as we move along the doping gradient. Variability in *V*_T_ between devices at fixed gate position with the same diameter (see Figure 4a) can be mostly attributed to processing variations leading to deteriorated precision in placement of the gate. Particularly, for thinner HSQ spacers, fluctuations in HSQ thickness constitute a larger absolute error of ±10 nm, attributed to the spacer fabrication method using underexposure of an electron beam resist. The HSQ thickness is evaluated by scanning electron microscopy (SEM) of the protruding nanowire during device fabrication; in addition, the thickness variation is determined by measuring the HSQ contrast curve using a profilometer (see the Device Fabrication section for details).^32,37^ Within the transitional region of the n^+^-/nid-segment, at *L*_HSQ_ = 50 nm, the spacer variation expectedly manifests as a larger variation due to steep change in *V*_T_ (Figure 3b). Notably, a doping concentration of 1 × 10^17^ cm^–3^ corresponds to only a few active Sn impurities within the channel region; thus, a variation in the Sn doping concentration due to the Au particle size and InAs nanowire diameter will have a large relative effect. Finally, we evaluate the charge density within the dielectric layer of the MOSFET gate-stack, corresponding to border traps *N*_bt_, by measuring the frequency dependence of maximum transconductance *g*_m,max_ in Figure 3c.^38^ Measurements are performed for radio frequency (RF)-optimized devices^39^ with gate-position *L*_HSQ_ at 50, 70, and 100 nm. Devices with thinner bottom spacer (*L*_HSQ_ = 10) nm are dominated by large gate-source overlap capacitance *C*_gs_ > 150 fF and are therefore unsuitable for RF analysis. The proposed gate-last fabrication method introduces direct gate-drain metal overlap, leading to a capacitance contribution of about *C*_gd_ ∼ 60 fF (calculated via small-signal modeling), sufficiently low to enable high-frequency measurements. Optimal bias conditions are obtained from DC measurements as *V*_DS_ = 0.5 V and *V*_GS_ corresponding to *g*_m,max_ (*V*_GS_ – *V*_T_ ≈ 0.25 V). For the MOSFETs with the lowest access resistance, i.e., the devices with the shortest HSQ thickness, we observe a very high transconductance reaching values close to 2 mS/μm, which is a competitive value considering the thin nanowire geometry. In agreement with planar III–V MOSFETs, the devices demonstrate the expected behavior of gradually increasing *g*_m,max_ with higher frequencies, up until parasitic capacitances (in conjunction with extrinsic resistance) dominate (>3 GHz). From these measurements, *N*_bt_ is calculated from the slope in the frequency range of 10^8^–10^9^ Hz, which yields similar border trap densities for all devices (∼10^19^ cm^–3^) independent of gate positions (see the Supporting Information for details). The results indicate that the oxide defects are not the main influence of the *V*_T_ shifts.

**Figure 5:**
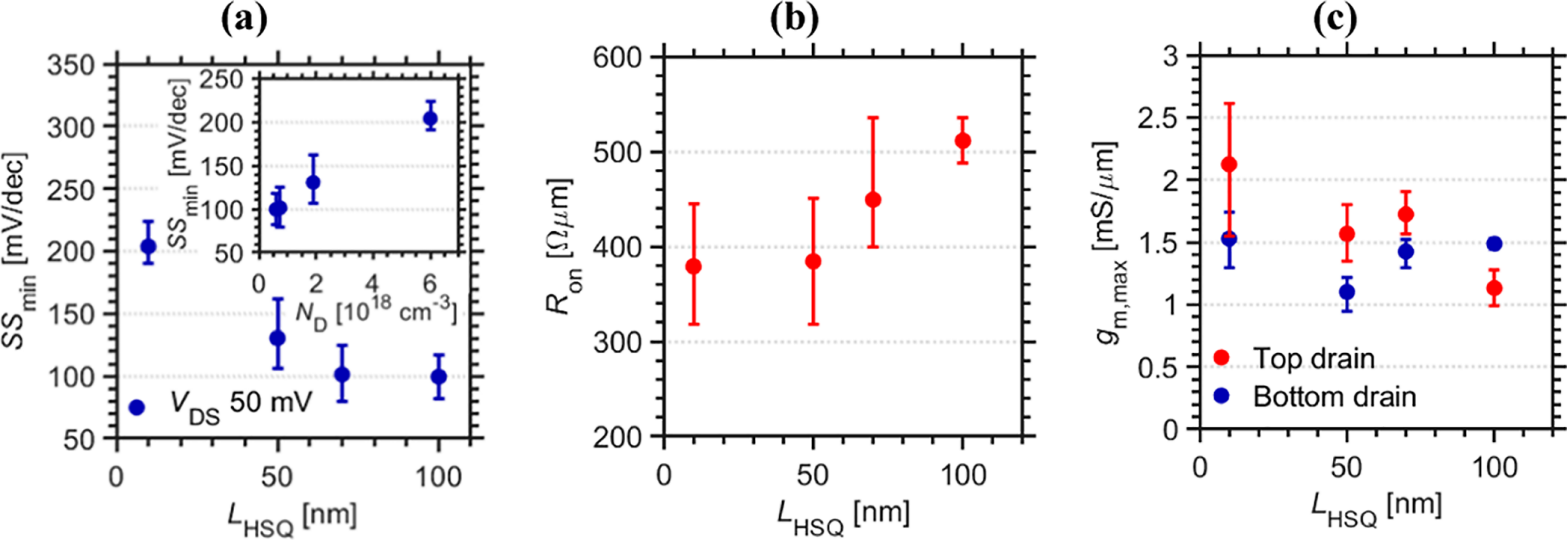
(a) SS_min_ behavior vs gate position *L*_HSQ_, with the inset correlating the gate position to expected channel doping *N*_D_ according to electron holography (Figure 3a). (b) On-resistance *R*_on_ vs *L*_HSQ_, demonstrating added series resistance with raised gate positions. (c) Maximum transconductance *g*_m,max_ dependence of gate position indicating degraded performance for larger *L*_HSQ_.

Finally, we evaluate the influence of the obtained doping profile (Figure 4a,b) of the InAs nanowire segment on relevant MOSFET performance metrics, such as minimum subthreshold swing SS_min_, on-resistance *R*_on_, and *g*_m,max_, with respect to gate position *L*_HSQ_ in Figure 5. Figure 5a provides off-state characteristics, quantified by SS_min_ (point slope) with respect to *L*_HSQ_, where increased channel doping leads to deteriorated off-state performance (figure inset). Improved SS_min_ for lower background doping is well in line with previously reported results for GAA InAs MOSFET devices.^40^ When moving the gate position up along the nanowire, the length of the epitaxial contact at the nanowire source (bottom) extends, resulting in an increased on-resistance *R*_on_; see Figure 5b.^35^*R*_on_ represents the combined resistance contribution of the transistor, namely, the sum of source and drain resistance (access resistance) as well as channel resistance. The maximum transconductance *g*_m,max_ of the devices shows state-of-the-art performance, with the best values exceeding 2.5 mS/μm, although they reduce with respect to raised gate position (Figure 5c). The DC performance of these 12 nm channel diameter devices, with *L*_g_ = 50 nm, compare well with previously reported vertical GAA MOSFETs, that demonstrated *g*_m,max_ > 3 mS/μm and *R*_on_ = 190 Ω·μm for devices scaled to *L*_g_ = 25 nm (17 nm channel diameter), albeit these devices provided less favorable off-state characteristics with reported SS_min_ = 440 mV/dec.^41^ The *g*_m,max_ vs *R*_on_ trends (Figure 5b) confirm the presence of an added ungated resistive regions at the source side for increased spacer thickness *L*_HSQ_.^33^ The added access resistance, at the source, also serves to reduce the effective voltage drop between the gate and source. This effect is predominantly observed at *L*_HSQ_ = 100 nm, as evidenced by increased transconductance values when switching the biasing of source and drain electrodes from bottom ground to top ground (Figure 5c). The presence of the axial doping distribution of the InAs nanowire core segment is therefore further validated by the trends measured for SS_min_, *R*_on_, and *g*_m,max_ vs gate position *L*_HSQ_.

## Conclusions

In conclusion, we have characterized the doping incorporation of Sn in ultrathin (12 nm channel diameter) vertical VLS-grown nanowires utilizing a novel method of axial threshold voltage probing validated by the well-established technique of high-resolution electron holography. The *V*_T_ probing method is performed by systematically moving the gate position along a vertical nanowire MOSFET and utilizing the measured shift in the threshold voltage to model and evaluate the encapsulated charge due to doping. By also measuring the transconductance–frequency dispersion, we further ruled out gate oxide defects as the main contribution for threshold voltage shift. The MOSFETs used in this study exhibited excellent performance, with highest maximum transconductance of 2.6 mS/μm. The obtained results are further substantiated by other transistor metrics, such as SS_min_, *R*_on_, and *g*_m,max_, which all scale according to gate position. This study also provided insights regarding the *V*_T_ variation typically found in III–V MOSFETs based on metal organic chemical vapor deposition (MOCVD)-grown materials, which has proven to be detrimental for further circuit implementation such as for CMOS applications. To address these issues related to in situ doping, the axial gradients could be mitigated by selective area epitaxy^42^ and further circumvented by employing regrown contacts.^43^ On a closing note, our proposed, *V*_T_-based, sweeping gate method allows characterization with sufficient resolution to discern various doping gradients present within thin nanowire channels employed in MOSFETs. The presented method, which requires no separately prepared samples, is therefore a welcome addition in the ever-growing library of application-specific devices employing advanced channel engineering.

## Methods

### Nanowire Growth

Arrays of Au disks with a thickness of 10 nm and diameters from 20 to 44 nm were patterned by EBL on substrates consisting of 250 nm highly doped InAs layers grown on high resistivity Si(111) substrates. The nanowires were grown using metal–organic vapor-phase epitaxy (MOVPE) in an Aixtron CCS 18313 reactor at a pressure of 100 mbar and a total flow of 8000 sccm. After annealing in arsine (AsH_3_) at 550 °C, an InAs segment was grown at 460 °C using trimethylindium (TMIn) and arsine with a molar fraction of *X*_TMIn_ = 6.1 × 10^–6^ and *X*_AsH_3__ = 1.3 × 10^–4^, respectively. The bottom and top parts of the InAs segment were n-doped by tetraethyltin (TESn) (*X*_TESn_ = 1.2 × 10^–5^). The growth was paused in an arsine flow for 3 min to reduce the In concentration in the Au particle. A 5 s pulse of TMIn and another 5 s pulse of trimethylantimony (TMSb) were supplied before a GaSb segment was grown using (TMGa) (*X*_TMGa_ = 4.9 × 10^–5^) and trimethylantimony (TMSb) (*X*_TMSb_ = 6.2 × 10^–5^) while heating to 515 °C. The top half of the GaSb segment was p-doped using diethylzinc (DEZn) (*X*_DEZn_ = 1.9 × 10^–5^). The temperature was then lowered to 460 °C at which a Sn-doped InAs shell was grown using the same molar fraction as for the InAs bottom segment.

### Electrical Measurements

DC measurements are realized with Cascade 1100B probe station connected to a Keithley 4200A-SCS parameter, where low-frequency RF probes are used to minimize access resistance originating from the probe-pad contact. RF measurements were carried out with an Agilent E8361A vector network analyzer. The measurement was calibrated off-chip with an LRRM method, and the effect of contact pads was deembedded by measuring dedicated on-chip open and short structures. *S*-parameters were measured from 10 MHz to 67 GHz and transformed to *y*-parameters. A small-signal model was fitted to the *y*-parameters, and the frequency dependence of the transconductance *g*_m,max_, as well as the defect density *N*_bt_, was calculated from Re(*y*_21_).

### TEM Analysis

Postgrowth nanowires are prepared by ozone oxidation followed by a 30 s HCl/H_2_O 1:10 dip (one digital etch cycle) to remove homogeneously doped radial shell growth. NWs were broken off from the growth substrate and transferred onto a TEM Cu grid with a carbon membrane. Electron holograms were recorded using an FEI Titan 80-300ST field emission gun transmission electron microscope, operating at 120 kV and equipped with a rotatable Möllenstedt biprism. This TEM technique of electron holography acquires a spatially resolved phase difference, ϕ, by interference between electrons that pass through the specimen (object wave) and vacuum (reference wave). The ϕ value is related to the crystal potential *V*(*x*, *y*, *z*) according to , where *C*_E_ is a constant that depends on the microscope acceleration voltage (8.64 × 10^–6^ rad/(m V) at 120 kV) and *t* is the specimen thickness. Holograms with 10 s exposure time were acquired using a 2 × 2-*k* charge-coupled device (CCD) camera (Gatan Ultrascan US1000 CCD) at a biprism voltage of 122 V. They were then processed through a removal of dead and hot pixels by an iterative local threshold algorithm, as well as a masking out of Fresnel fringes. In addition, to increase the signal-to-noise ratio of the holograms, a modest Wiener filtering in Fourier space was employed. Upon the holographic Fourier reconstruction method, one side band was masked with a circular 10th-order Butterworth filter. Finally, the phase of the reconstructed wave was subtracted by the phase reconstructed from an additionally recorded and equally processed object-free empty hologram. The mean counts per hologram pixel were ≈60 000, the phase resolution was ≈0.2 rad, and the error in the potential was ≈0.09 V.

### Device Fabrication

The sample was first spin-coated with hydrogen silsesquioxane (HSQ) thin film and patterned via e-beam lithography (EBL), where the local spacer thickness is controlled by the dose of electrons. After development of the HSQ film by a 25% tetramethylammonium hydroxide (TMAH) solution, the sample was dipped in citric acid followed by 20 nm sputtered W and 3 nm atomic layer deposited (ALD) TiN. A C4F8:Ar anisotropic dry etch was performed, which leaves only the metal on the nanowire sidewalls, forming the top contact.

The exposed HSQ was then thinned by diluted HF 1:1000 to form the first spacer, exposing the channel region, forming a recess gate. This allows for selective etching of the gate by digital etching, namely, repeated oxidization with O_3_ and etching by citric acid until the highly doped InAs shell is removed. In situ hydrogen-plasma cleaning is performed prior to high-*k* deposition, at 250 degrees, consisting of 40 cycles of HfO_2_. The gate was then finalized by sputtering 60 nm of W, which is vertically aligned by a back-etched polymer spacer, by O_2_-plasma, followed by an SF6 dry etch for W removal. Postmetal annealing at 250 °C is performed after gate deposition. The MOSFET is then finished by aligning an S1813 top spacer, forming via holes, and sputtering of Ni/W/Au, 15/30/180 nm, as the final top metal.

## Supporting Information Available

MOSFET virtual source modeling, VT dependence of nanowire diameter (quantization considering nonparabolicity), response of boarder traps with respect to oxide depth derived from RF measurements, and expanded dataset of transfer characteristics with respect to gate position (PDF)
