# Supplementary material for: Doping Profiles in Ultrathin Vertical VLS-Grown InAs Nanowire MOSFETs with High Performance
Source: ACS Appl Electron Mater. 2021 Nov 19;3(12):5240–7. doi: 10.1021/acsaelm.1c00729 (PMC8717821; doi:10.1021/acsaelm.1c00729)
Supplement: Supplementary file 1 — el1c00729_si_001.pdf [file el1c00729_si_001.pdf]

Supporting Information

Doping Profiles in Ultrathin Vertical VLS-Grown InAs Nanowire MOSFETs with High Performance

Adam Jönsson* [>], Johannes Svensson[>], Elisabetta Maria Fiordaliso[>>], Erik Lind[>], Markus Hellenbrand[>>>], and Lars-Erik Wernersson[>]

[>]**Department of Electrical and Information Technology, Lund University, Box 118, 221 00 Lund, Sweden**

[>>]**National Centre for Nano Fabrication and Characterization, Technical University of Denmark, Fysikvej 307 & 126, 2800 Kongens Lyngby, Denmark**

[>>>]**Department of Material Science & Metallurgy, University of Cambridge, 27 Charles Babbage Road, CB3 0FS Cambridge**

E-mail: Adam Jönsson adam.jonsson@eit.lth.se, Johannes Svensson Johannes.svensson@eit.lth.se, Elisabetta Maria Fiordaliso emfi@dtu.dk, Erik Lind erik.lind@eit.lth.se, Markus Hellenbrand mkhh2@cam.ac.uk, Lars-Erik Wernersson lars-erik.wernersson@eit.lth.se

This work was supported in part by the Swedish Research Council, in part by the Swedish Foundation for Strategic Research, and in part by the European Union H2020 Program INSIGHT under Grant 688784.

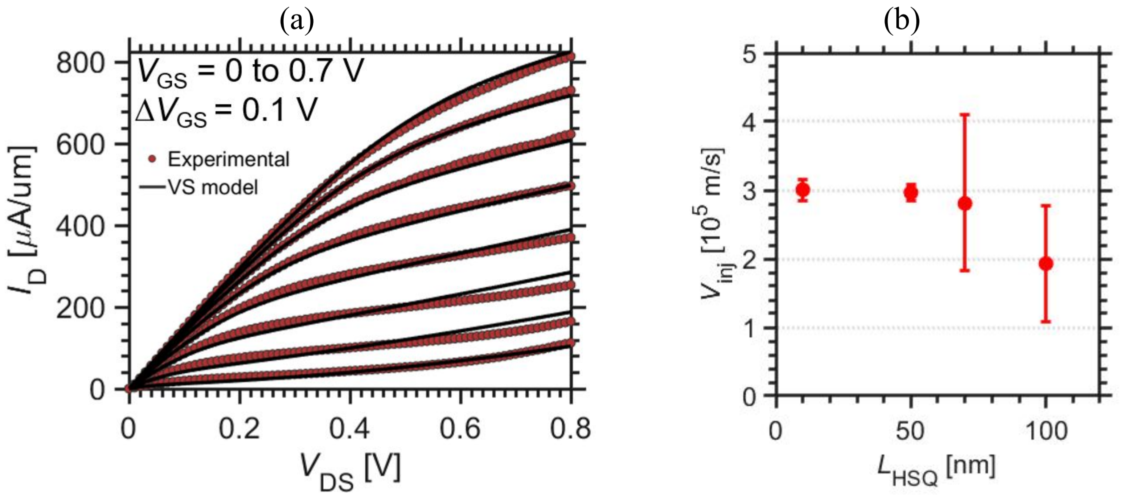

Figure S1. (a) Output characteristics of a single InAs MOSFET with fitted virtual source model according to guidelines in [1]. The virtual source model is semi-empirical employing physical parameters, where fitting is done by three main metrics: injection velocity (v_{inj}), low-field mobility and access resistance. For device operating in saturation the normalized (by gate-width W) current I_D is described as $I_D/W = Q_{x0}v_{inj}$, where ballistic device behavior is captured by amount of charge carriers Q_{x0} available at the source-side adjacent to the gate and their associated injection velocity v_{inj}. (b) Extracted injection velocity by virtual source model for devices with varied gate-position L_{HSQ}. Injection velocities around $3{\cdot}10^5$ m/s are derived for most of the MOSFETs, corresponding well with previously reported values for vertical GAA MOSFETs (InAs source) at 50 nm gate-length. [2] Constant injection velocity are expected for devices with similar gate-length assuming a quasi-ballistic transport translating to constant transmission conditions.

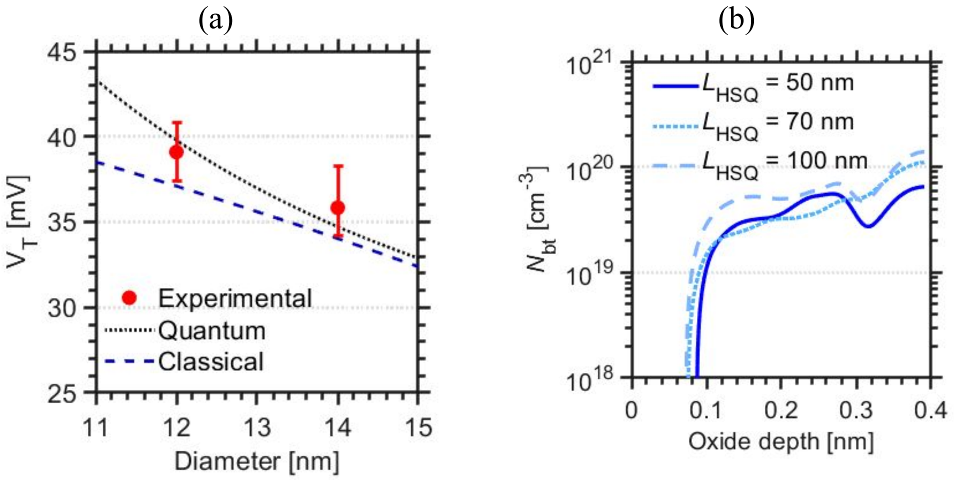

Figure S2. (a) Comparing devices at similar gate-position (L_{HSQ} = 70 nm) for two different diameters. To evaluate potential impact of quantization effects due to diameter variation in comparison to the doping, we calculate the energy shift E_{11} in the first sub-band within the nanowire as described by $E_{11} = \sqrt{1 + \alpha\hbar^2\pi^2/(2r^2m^*)} - 1)/(2\alpha)$, see dotted line, where α represent a non-parabolicity factor, and m^* the bulk effective mass [3]. Calculated quantization is based on hard wall confinement, and it displays a small V_T shift of 7 meV for an 11 to 15 nm diameter range. The shift in the cylindrical geometry is small as compared to thinner lateral nanowire sheet structures (8 nm height) where a variation over 100 mV has been reported [4]. We note that the cylindrical geometry is advantageous for scaling as it is less sensitive to size variations, thus we conclude that the diameter variation in our devices has a relative minor effect on the voltage shift compared to channel doping. (b) By measuring the maximum transconductance $g_{m,max}$ vs frequency dispersion, the N_{bt} can be deduced from the slope in a medium frequency range prior to roll-off induced by extrinsic capacitances at the highest frequencies. De-embedding is performed via a small signal model in order to determine the intrinsic transconductance whose frequency behavior translates to the trap response. [5] When assuming elastic tunneling, the depth of the trap states within the oxide can be associated with different time constants τ and can be converted to depth into oxide Z_t according to $Z_t = \lambda \ln \frac{\tau}{\tau_0}$. Here τ_0 represents the tunneling time constant for a trap state at the semiconductor/oxide interface and λ the attenuation coefficient (calculated to 0.13 nm) of the wave function penetrating the oxide. From the data, it is clearly indicated that the N_{bt} is independent of the position along the nanowire and thus also the doping inside the channel material, namely the oxide quality is not significantly affected by dopant atoms. The estimated N_{bt} is in line with our previous results using different characterization techniques for III-V MOSFET devices, which are typically about an order of magnitude higher compared to state-of-the-art Si devices. [5,6]

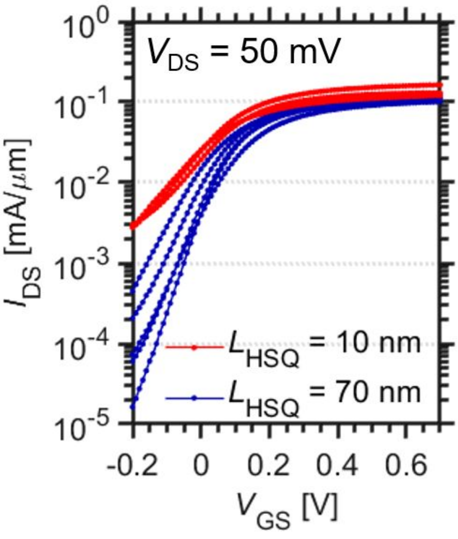

Figure S3. Transfer characteristics at V_{DS} = 50 mV highlighting differences in behavior between doped and non-intentionally doped (nid) channel regions, at L_{HSQ} = 10 nm and 70 nm respectively (5 devices represented for each gate-position). Dataset with respect to L_{HSQ} = 10 nm (highly doped channel) shows coherent results with overlapping data, although transfer characteristics L_{HSQ} = 70 nm shows significant spread in the off-state region. This is expected due to gate-placement within transitional n+/nid-region. A clear trend with respect to V_T-shift can be discerned between the various gate-placement, as well as significant increase in off-state current due to diminished electrostatic control for a highly doped channel.

References

1. Khakifirooz, A., Nayfeh, O. M. & Antoniadis, D. A Simple Semiempirical Short-Channel MOSFET Current–Voltage Model Continuous Across All Regions of Operation and Employing Only Physical Parameters. *IEEE Trans. Electron Devices* **56**, 1674–1680 (2009) doi:10.1109/TED.2009.2024022.

2. Kilpi, O., Svensson, J., Lind, E. & Wernersson, L. Electrical Properties of Vertical InAs / InGaAs. *IEEE J. Electron Devices Soc.* **7**, 70–75 (2019) doi:10.1109/JEDS.2018.2878659.

3. Lind, E. High frequency III – V nanowire MOSFETs. *Semicond. Sci. Technol.* **31**, 1–13 (2016) doi:10.1088/0268-1242/31/9/093005.

4. Zota, C. B. & Lind, E. Size-effects in indium gallium arsenide nanowire field-effect transistors. *Appl. Phys. Lett.* **109**, 063505 (2016) doi:10.1063/1.4961109.

5. Johansson, S., Berg, M., Persson, K. M. & Lind, E. A high-frequency transconductance method for characterization of high-k border traps in III-V MOSFETs. *IEEE Trans. Electron Devices* **60**, 776–781 (2013) doi:10.1109/TED.2012.2231867.

6. Hellenbrand, M., Kilpi, O.-P., Svensson, J., Lind, E. & Wernersson, L.-E. Low-frequency noise in nanowire and planar III-V MOSFETs. *Microelectron. Eng.* 110986 (2019) doi:10.1016/J.MEE.2019.110986.
